# Pyelonephritis in cats and dogs - a scoping review

**DOI:** 10.1186/s12917-026-06042-1

**Published:** 2026-10-26

**Authors:** L. Pelander, C. Ekstrand, K. Scahill

**Affiliations:** 1 Department of Clinical Sciences, Swedish University of Agricultural Sciences, Ulls Vag 26, Uppsala, 75007 Sweden; 2 Department of Animal Biosciences, Swedish University of Agricultural Sciences, Ulls Vag 26, Uppsala, 75007 Sweden; 3 International Veterinary Evidence-Based Guidelines Centre, Department of Veterinary Clinical Science, University of Copenhagen, Frederiksberg, C 1870 Denmark; 4 Evidensia Södra Djursjukhuset, Månskärarvägen 13, Huddinge, 141 75 Sweden

**Keywords:** Canine, Feline, Urinary tract infection

## Abstract

There is a lack of consensus regarding the diagnosis and treatment of bacterial pyelonephritis (PN) in dogs and cats. The aim of this scoping review was to increase knowledge by describing published scientific literature on the subject, specifically to collate definitions and diagnostic criteria, antimicrobial regimens, reported outcomes, and to identify knowledge gaps. A structured search was performed of the bibliographic databases Biosis, CAB Abstracts, Medline, Web of Science Core Collection and Scopus. We included 176 original studies written in English or German that reported on any aspect of spontaneous PN (risk factors, signalment, history, clinical findings, diagnostic criteria, treatment, prognosis, and outcome) in cats or dogs. Three types of PN were identified in the literature: histologically diagnosed PN, clinically diagnosed PN, and pyonephrosis. Multiple information gaps regarding clinical aspects of PN in dogs and cats were detected. There were no universally accepted clinical criteria for clinical diagnosis of PN and no clinical trials evaluating antimicrobial or other treatment options. Information about short-term outcomes for dogs and cats with PN was scarce, and there were no data regarding long-term outcomes. More information regarding all aspects of clinical PN in dogs and cats is needed.

The study protocol was registered prospectively in 2023 (https://syreaf.org/protocols/).

**Supplementary Information:**

The online version contains supplementary material available at https://doi.org/10.1186/s12917-026-06042-1.

## Introduction

Bacterial pyelonephritis (PN) is usually caused by ascending bacterial infection of the kidney, though a haematogenous route is also possible. It represents a potentially life-threatening, but often treatable condition [1]. Both dogs and cats are affected, and the condition may be acute or chronic. Substantial uncertainty exists regarding both diagnosis and optimal treatment in both species. Especially chronic forms of PN represent extraordinary diagnostic challenges for clinicians because of a frequent lack of localising signs of infection. To the authors’ knowledge, no specific and universally accepted clinical definition of (diagnostic criteria for) PN in dogs and cats exists, further complicating diagnosis. For establishment of a clinical diagnosis of PN, physical examination findings and clinicopathological data are often combined with imaging of the urinary tract. Pyelonephritis is often considered a differential diagnosis in dogs and cats with renal pelvic dilation, which can also result from diuresis, ureteral obstruction, or chronic kidney disease without bacterial infection [2]. Consequently, both over- and underdiagnosis of PN are likely to be relatively common, negatively affecting patient care. Fluoroquinolones are recommended internationally for treatment of PN in both species [1]. These antibacterial substances are critically important in human medicine, and their use in animals should be restricted to mitigate the risk to the public, according to the European Medicines Agency. This represents one important reason to keep overdiagnosis of PN to a minimum. Evidence-based recommendations regarding diagnosis, choice of antibacterial substance, and duration of treatment are presently difficult to make because of a lack of high-quality evidence to answer those clinical questions.

It is important that the most relevant outcomes, those that matter to owners, patients, and clinicians, are measured in clinical studies to determine the most effective treatment and management strategies for a particular disease [3]. Different variables are often used among studies, and this limits the evidence available to help with the clinical decision-making process. Knowledge about outcomes used in previously published studies on PN in cats and dogs might be of help in future establishment of core outcome sets (agreed set of outcomes to be measured and reported as a minimum in future studies conducted relating to a particular disease). It is unclear what information is available in the literature regarding PN in dogs and cats, and because of the existence of a broad range of unanswered clinical questions regarding definition, diagnosis, treatment and monitoring, and a perceived lack of studies in the area, a scoping review was considered the most appropriate knowledge synthesis method.

## Materials and methods

The aim of this scoping review was to summarise current knowledge regarding the diagnosis and treatment of PN in dogs and cats. The objectives were to describe the volume, nature, and characteristics of published scientific literature on PN in dogs and cats, and to collate case definitions and diagnostic criteria, antibacterial regimens, and reported outcomes. A secondary objective was to identify knowledge gaps and prioritise future research.

### Protocol and registration

The protocol for this scoping review was drafted using the Preferred Reporting Items for Systematic Reviews and Meta-Analysis for Scoping Reviews (PRISMA-ScR) reporting guidelines [4]. The final protocol was registered prospectively with SYREAF, Systematic Reviews for Animals & Food in March 2023 [5]. No substantial deviations were made from the protocol. The study was reported according to the PRISMA ScR and the PRISMA 2020 updated guideline for reporting systematic reviews [6]. The PRISMA checklist is provided as additional file 1.

### Information sources, search and initial screening

Search strategies were drafted by the authors and two information specialists at the Swedish University of Agricultural Sciences. The bibliographic databases Biosis, CAB Abstracts, Medline (Web of Science interface), Web of Science Core Collection and Scopus were searched, as recommended [7]. There were no initial restrictions for language, year of publication, or publication type. Review texts published < 10 years of the initial search were used to screen reference lists for eligible studies. No supplementary attempts to reach grey literature were made. The initial search was performed on the 22nd of March 2023, and a second search was performed on the 5th of February 2025 to include more recent publications. Detailed information about the search process is provided in additional files 2 and 3. References in chapters relevant to PN in the most recent editions of four companion animal medicine textbooks were also reviewed [8–11].

Individual database results were imported into EndNote X9.3.3 (Clarivate) software, where duplicates were removed using the Karolinska Institute University library method [12]. Thereafter, the final reference list was imported into Rayyan, an internet-based program [13], for blinded screening. The updated screening in 2025 was conducted in Covidence [14]. Three authors (LP, KS, CE) took part in the identification of potentially eligible studies from the search based on titles and abstracts. Full texts were retrieved whenever there was uncertainty about eligibility from review of title and abstract only.

### Eligibility criteria

Publications remaining after initial screening were retrieved, and full texts were reviewed independently by two reviewers (LP, KS). To be eligible for inclusion in the review, studies had to be written in English or German and report on any aspect of spontaneous PN (risk factors, signalment, history, clinical findings, diagnostic criteria, treatment, prognosis, and outcome) in cats or dogs. Studies of acute kidney injury (AKI) in dogs and cats were included if infection was the reason for AKI in any of the animals. Urinary tract infection studies were included unless clearly stated that the infections were situated in the lower urinary tract (cystitis, prostatitis, urethritis). Case reports were included. Publications were excluded if animals were experimentally infected or infected with Leptospira species. Studies in people were excluded.

### Data extraction

A Microsoft Excel data extraction sheet was constructed for documentation of relevant information from included studies. Two authors independently extracted data from all references, and any inconsistencies were resolved by collective agreement. An initial pilot was conducted on 20 references, after which the form was refined. Given the heterogeneity of included sources, the pilot was expanded to 40 references to ensure that the form adequately captured variation in study design, source type, terminology, and reporting detail. Following this process, the final data charting form was applied to all included sources. The data extracted included study characteristics (title, journal, year of publication, study type) and population characteristics (species, number of animals with PN). Also, diagnostic criteria for the diagnosis of PN (if present), clinical data, treatment, and outcome data were recorded. Studies were characterised as clinical trials, observational studies, or case reports. Studies that reported clinical cases case by case were defined as case reports irrespective of the number of cases described.

## Results

### Included studies and study characteristics

**Fig. 1:**
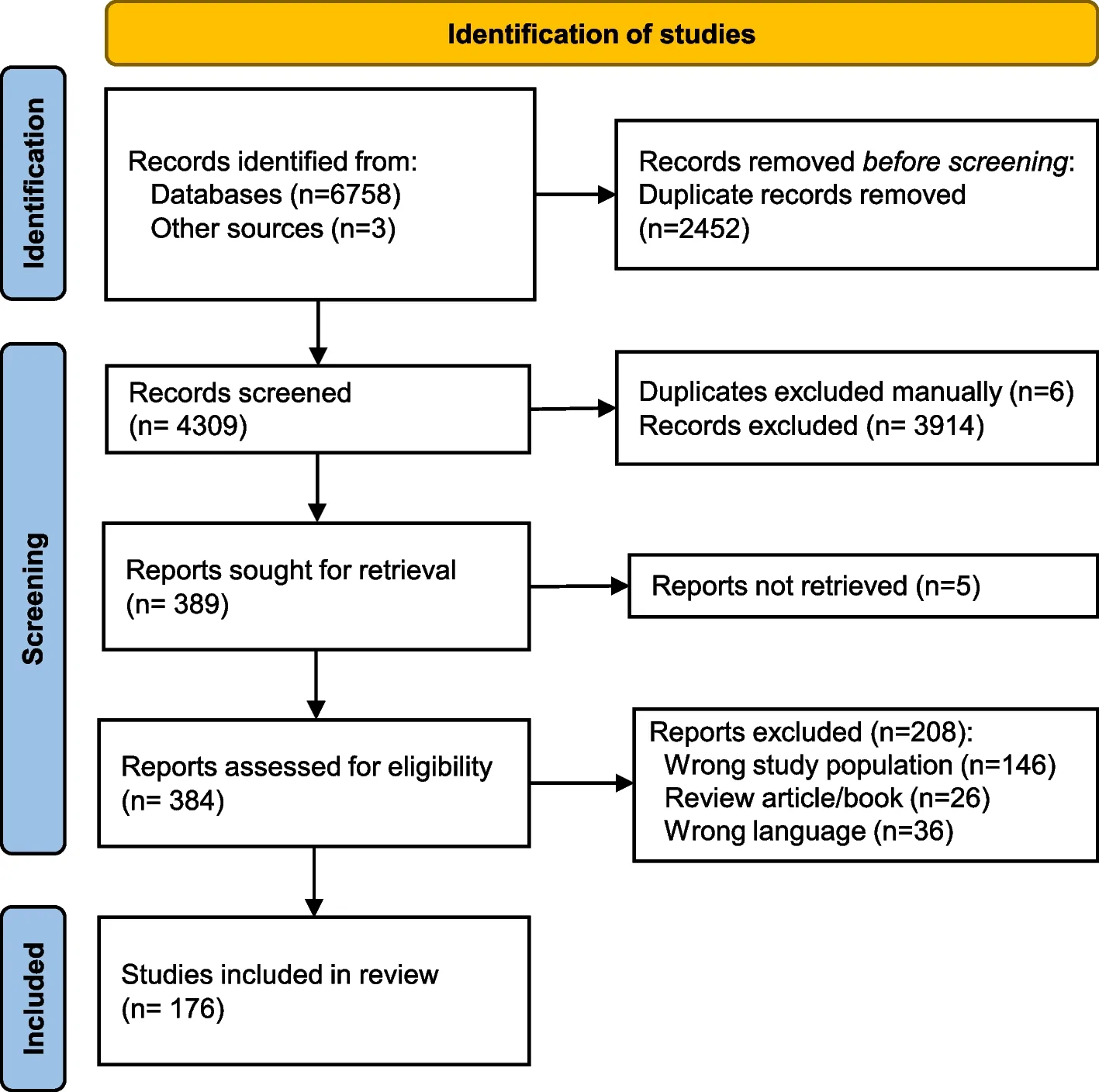
Flow diagram of search and screening process

The initial search resulted in the identification of 6443 references. After removal of duplicates, the number of unique references identified in the first search was 3994. The second search identified a further 315 unique studies, making the total number of screened studies (title and abstract) 4309. Figure 1 shows the search and study identification process in detail.

**Fig. 2:**
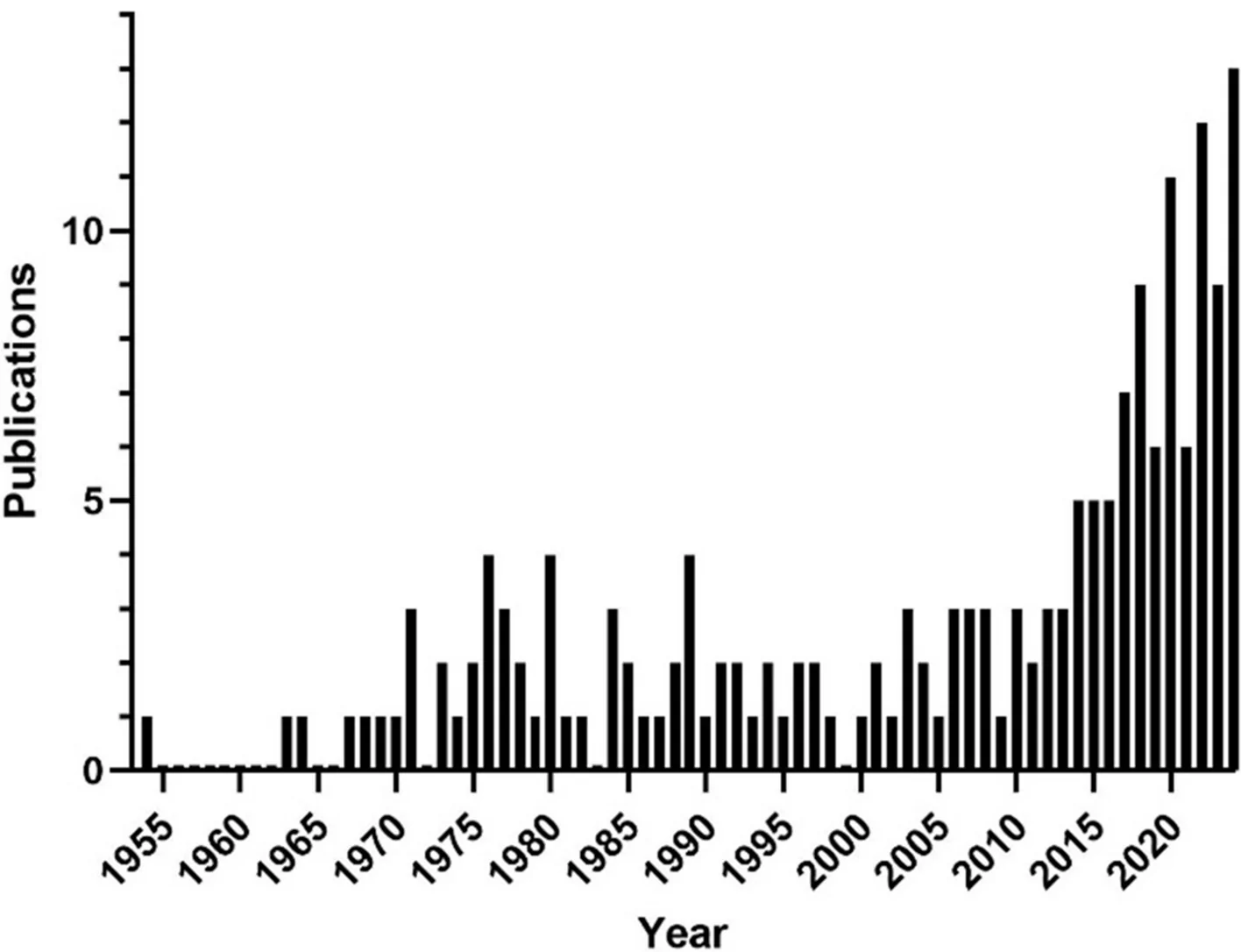
Publication year for the 176 studies included in this review

One hundred and seventy-six studies involving approximately 1,600 cats and dogs with PN were included for data extraction [2, 15–189]. Of these animals, 817 were identified in two retrospective studies evaluating prescription data in dogs and cats diagnosed with upper or lower bacterial urinary tract infections at several veterinary practices in the USA and Canada [180, 181]. One hundred and fifty-five studies concerned dogs, 55 studies concerned cats, and 6 studies concerned both species. The studies included in this review were published between 1954 and 2025, with an increase in publication numbers in the last 10 years (Fig. 2). Most studies were conducted in the US (*n* = 60), followed by Germany (*n* = 19), India (*n* = 15), the United Kingdom (*n* = 14), Australia (*n* = 11), Canada (*n* = 10), and Korea (*n* = 10). The remaining 37 studies were performed in 14 other countries. The journals with the highest number of included studies were Journal of the American Veterinary Medical Association (*n* = 27), Journal of Veterinary Internal Medicine (*n* = 23), Journal of Feline Medicine and Surgery (*n* = 10), and Journal of Small Animal Practice (*n* = 9). The remaining 107 studies were published in 52 other journals. There were 95 observational studies (Tables 1 and 2) and 81 case reports (Table 3). Observational studies that used histopathological criteria for the diagnosis of PN (*n* = 18) and 4 studies that described pyonephrosis (accumulation of purulent material in the renal pelvis secondary to an obstruction) are reported separately in Table 2. No clinical trials were identified, but one meta-analysis investigated mortality in dogs and cats with AKI based on aetiology (listed among observational studies in Table 1). The meta-analysis suggested higher rates of overall mortality in cats and dogs with AKI due to noninfectious aetiologies relative to infectious aetiologies.

**Table 1:** Study characteristics table for observational studies of pyelonephritis in dogs and cats

Author	Year	Country	Sp	n	Specific objective(s)	Cr^1^ Y/N	Data on clinical outcomes^2^
Bar-Nathan	2022	Israel	D	1	To determine the long-term outcome of dogs recovering from AKI, assess the proportion of dogs with normalization of sCr at discharge and during the follow-up period, and identify prognostic factors for both normalization of sCr and long-term outcome	Y	None
Beaston	2023	USA	C	2	To describe POCUS findings in azotaemic cats with ureteral obstruction	N	None
Bendeler	2023	Switzerland	D	13	To investigate the prevalence of increased DGGR-lipase activity in dogs with AKI treated with and without HD, and to evaluate the clinical relevance of hyperlipasemia and its association with severity of kidney disease, treatment modality, and outcome	N	None
Berent	2014	USA	C	1	To evaluate the incidence of mineralisation, infections, dysuria, and azotaemia when tEDTA was routinely infused into each SUB, compared to a saline infusion control group	N	None
Bhojne	2022	India	D	43	To describe ultrasonographic findings in dogs with renal disease	N	None
Biscop	2024	Belgium	D	1	To evaluate uTIMP-2, uIGFBP7, and SDMA in healthy dogs, dogs with CKD, dogs with AKI, and CI dogs. To assess the potential of NGAL in differentiating dogs with AKI from CKD and CI dogs	N	None
Brady	2000	USA	C	2	To document the clinical, clinicopathologic, and pathologic findings in cats with severe sepsis and to identify abnormalities unique to this species. To identify criteria that could be used antemortem to diagnose SIRS in cats	N	None
Broughton	2023	UK	C	2	To describe cats with ionised hypercalcemia in a referral centre and to describe the biochemical and signalment differences among causes of ionised hypercalcemia. To identify the proportion of cases with each diagnosis that had concurrent urolithiasis	N	None
Bruneau	2024	USA	C	3	To describe the patient population, sites of infection, targeted bacterial isolates, chloramphenicol dosages, treatment durations, outcomes and adverse effects in cats treated with chloramphenicol	N	CC, BC, decrease in azotaemia
Choo	2022	Korea	D	1	To determine whether there is a significant difference in actual and predicted RCT, considering the BW and BCS of dogs with acute or chronic renal disease. To assess the usefulness of the RCT:Ao ratio as a quantitative index reflecting renal pathology in dogs with acute and chronic renal disease	N	None
Cole	2017	UK	C	3	To describe the prevalence of SH in cats with AKI and to describe its relationship with disease severity and outcome	N	None
Cole	2020	UK	D	3	To describe the prevalence of SH and hypertensive retinopathy in dogs with AKI. To investigate the relationship between SH and severity of AKI and to assess possible factors associated with SH	N	None
Crisp	1989	USA	D C	2	To determine the value of the procedure and complications encountered in animals treated with peritoneal dialysis for acute or chronic renal failure	N	None
D Ánjou	2011	Canada	D C	16	To determine the maximal width of the renal pelvis in dogs and cats with clinically normal renal function, with and without increased diuresis, and to compare these values to those obtained in animals with nonobstructive urinary tract disorders and those with outflow obstruction. To determine whether a cut-off value could be used to predict obstruction in this population	Y	None
Decome	2020	Canada	D	3	To describe the in vitro antimicrobial susceptibility of P. *mirabilis* and its evolution over the study period, describe the clinical significance of identification of P. *mirabilis* bacteriuria, and to identify risk factors in dogs with P. *mirabilis*-associated UTIs	Y	None
Degenhardt	2023	Germany	C	4	To investigate SAA concentrations in cats with azotaemic kidney disease to determine whether SAA concentration can be used to differentiate between AKI, CKD, and AoC. To evaluate whether SAA concentration can be used as a prognostic parameter. To determine the correlations of SAA with temperature; creatinine, urea, and albumin concentrations; leucocyte count; and urine protein/creatinine (UP/C)	N	None
Deprey	2021	France	C	1	To report the incidence and risk factors associated with positive intraoperative and postoperative urine cultures and to determine the long-term outcome in cats with subcutaneous ureteral bypass	N	None
Dorval	2009	Canada	C	2	To describe the application, efficacy, and clinical features of cats treated with PD for ARF of different aetiologies	N	Survival to discharge
Dunaevich	2020	Israel	D	15	To characterise the causes, clinical signs, laboratory results, and the short- and long-term prognosis of dogs with ACKD	Y	Survival to discharge
Dörfelt	2007	Germany	D	1	To evaluate the technical feasibility of haemodialysis and its efficacy and complications in dogs with acute renal failure	N	Euthanasia
Eatroff	2012	USA	D C	1*	To determine the long-term outcome, defined as a survival time of 365 days or longer, of cats and dogs treated with IHD for AKI. To examine whether any variables that influenced survival time could be identified	N	None
Elbert	2022	USA	D	4	To describe the neuropathologic features of systemic bacterial infection in a retrospective series of 28 dogs	N	Survival to discharge
Fischer	2023	Germany	D	1	To evaluate primary, secondary, and tertiary haemostasis in azotaemic dogs using viscoelastic testing, compare them to those in healthy dogs, and examine the association between changes in haemostasis and mortality. To investigate differences in haemostatic parameters between dogs with CKD and those with AKI	N	None
Foster	2018	USA	D	14	To retrospectively evaluate the prevalence of bacteriuria in a population of dogs with CKD; to further categorise these patients as having SBU, bacterial cystitis, or pyelonephritis; and to summarise bacterial isolates commonly identified by microbial culture of urine samples from these dogs	N	Mortality < 1 month
Frey	2024	USA	D	1	To provide additional insight into clinical and microbial outcomes in dogs with untreated SB by identifying dogs with SB, conducting longitudinal follow-up, and characterizing these outcomes	N	Euthanasia (24 h)
Godia	2022	Spain/Denmark	C	10	To characterise the feline urinary miRNAome by profiling miRNAs in urine using small RNA sequencing. To identify a panel of differentially abundant urinary miRNAs in cats with PN and cats with other urological diseases using qPCR, to establish discriminatory abundance profiles for diagnostic use	Y	None
Gookin	1996	USA	D	5	To evaluate indications for and complications, efficacy, and effects on renal function of unilateral nephrectomy in dogs with renal disease, and to evaluate the role that scintigraphy had in the decision to excise a kidney	Y	Survival time
Jessen	2019	Denmark	C	5	To investigate the presence and stability of miRNAs in whole urine of cats. To investigate if selected miRNAs are differentially expressed in cats with PN compared to healthy cats and cats with CKD, UO, or SB	Y	None
Johnson	2024	USA	D	10	To assess the perioperative and long-term complication rate associated with unilateral nephrectomies in dogs with renal disease and assess for factors that may increase the risk of CKD development	N	None
Kennedy	2021	Australia	C	1	To evaluate the potential association between husbandry factors, specifically diet, and UO in cats	N	None
Kern	2018	USA	D	5	To describe the populations of dogs with E. *coli* UTI that do and do not exhibit biofilm-forming capability. To compare the clinical characteristics of dogs with biofilm-forming E. *coli* bacteriuria to dogs with nonbiofilm-forming E. *coli* bacteriuria	Y	None
Kogika	1995	Brazil	D	13	To study the prevalence of some microorganisms in dogs with UTI and their antimicrobial susceptibility	N	None
Kohn	2006	Germany	C	1	To evaluate underlying diseases in cats with thrombocytopenia and to establish possible causes of platelet deprivation	N	None
Kopecny	2019	USA	C	8	To identify risk factors associated with positive urine cultures in cats undergoing ureteral stent and SUB placement to relieve benign ureteral obstruction. To report the incidence of subclinical bacteriuria and UTI in these cats	Y	None
Kulendra	2021	UK	C	4	To evaluate factors affecting the outcome of cats that had SUBs placed secondary to benign ureteral obstruction and describe any complications encountered	N	None
Kurtz	2024	France	C	17	To report and compare the clinical, biological, and diagnostic imaging findings in a cohort of cats with suspected or confirmed bacterial pyelonephritis. To evaluate the accuracy of SAA concentration as a marker of bacterial pyelonephritis in cats	Y	None
Lábee Lund	2003	Norway	D	1	To characterise the mycoplasmas isolated and to elucidate the association between them and the clinicopathological findings in the dogs	N	Euthanasia
Lamb	1998	UK	D	2	To evaluate ultrasonography as an alternative to contrast radiography for diagnosis of ectopic ureter in dogs	N	None
Langston	1997	USA	C	6	To investigate the application, efficacy, and clinical consequences of haemodialysis in cats with life-threatening uremia	Y	Survival, discontinuation of HD
Legatti	2018	Brazil	D C	20	Meta-analysis. To evaluate the incidence of mortality in cats and dogs with AKI based on aetiology (i.e. infectious versus noninfectious; receiving dialysis versus conservative treatment)	N	None
Lehner	2020	Switzerland	D	19	To evaluate whether there is a change in antimicrobial prescriptions to dogs with acute diarrhoea, urinary tract infection, respiratory tract infection, and wound infection by Swiss veterinarians after the introduction of the online ASP tool AntibioticScout.ch	N	None
Ling	1985	USA	D	6	To determine whether the antibiotic-coated bacteria test could be used to indicate bacterial invasion of uroepithelium and/or the site of infection in female dogs with naturally occurring UTI	Y	None
Loane	2022	UK	C	3	To evaluate serum SDMA concentrations in cats with azotaemic AKI, CKD and controls to assess its suitability as a biomarker for AKI	Y	None
Manucci	2019	Italy	D	2	To investigate whether contrast-enhanced ultrasonography can evaluate the changes in renal perfusion in dogs with AKI	N	None
McGovern	2019	USA	D	10	To evaluate the spectrum of bacteria involved in UTI and their susceptibility to antimicrobials, including multidrug resistance, clinical parameters, and results of urine sediment analysis	Y	None
Mukherjee	2014	India	D	5	To examine the load of pathogenic Staphylococci in different urinary tract infections of canines, followed by their characterisation, pathogenicity testing, and antibiotic sensitivity testing, to find suitable drugs against these pathogens	N	None
Neumann	2024	Canada	D	4	To assess the short- and long-term outcome of artificial urethral sphincter placement in male dogs with urethral diverticulum	N	None
Perondi	2018	Italy	D	2	To evaluate the prognostic accuracy of three scoring models and the presence of pancreatitis and systemic inflammatory response syndrome in predicting outcome in a cohort of dogs with AKI and AKI/CKD managed by haemodialysis	N	None
Perry	2022	USA	D	16	To describe the clinical characteristics and outcome of a cohort of dogs with urosepsis. To determine if MODS occurred in some dogs with urosepsis and whether the number of organs affected was associated with their mortality rate. To assign the severity score APPLEfast and compare it to patient outcome	Y	Survival to discharge
Puccinelli	2023	Japan	D	1	To evaluate the feasibility and diagnostic value of two-dimensional shear wave elastography (2D-SWE) in dogs with AKI, CKD, and AKI/CKD and its correlation with renal functional and prognostic parameters (urinary output, creatinine, urea, serum calcium-phosphorus product). To evaluate the possible correlations between 2D-SWE values and contrast-enhanced ultrasound, and to investigate the possible influence of renal perfusion modifications on renal stiffness	N	None
Quimby	2017	USA	C	13	To determine ultrasonographic characteristics of the renal pelvis and proximal ureter of cats with stable chronic kidney disease and determine if these were significantly different from cats with pyelonephritis and ureteral obstruction	Y	None
Ramakant	2013	India	D	3	To compare ultrasonographic findings in healthy and renal failure dogs	N	None
Rimer	2022	Israel	D	3	To characterise the aetiology, clinical and clinicopathologic findings, and outcome in a large cohort of dogs diagnosed with AKI and (b) identify risk factors for death	Y	None
Saeki	2015	Japan	D	3	To evaluate the feasibility and clinical outcome of total cystectomy and urinary diversion to the prepuce or vagina for the treatment of TCC involving the trigone of the bladder	N	None
Sallam	2022	Egypt	C	2	To record the common surgical disorders of the urogenital system in cats admitted to private pet animal clinics in Alexandria	N	None
Sapin	2016	Brazil	D	14	To classify qualitatively and quantitatively the urinary tract lesions of 363 dogs between 1978–2014 at the Universidad Federal de Pelotas	N	None
Sato	2002	Japan	D	2	To record the common surgical disorders of the urogenital system in cats admitted to private animal clinics in Alexandria	N	None
Schalm	1970	USA	C	4	To ascertain the percentage distribution of plasma fibrinogen levels in cats, dogs, horses, and cattle	N	None
Segev	2013	USA	C	7	To assess clinical severity of AKI in cats presenting with acute uremia and managed with intermittent haemodialysis. To characterise clinical signs, clinicopathologic abnormalities, common aetiologies, and outcomes of these same cats to develop clinically applicable predictive models	N	Survival
Shin	2020	Korea	C	1	To evaluate the efficacy of double-pigtail ureteral stent fixation to reduce stent migration	N	None
Sinha	1977	India	D	12	To describe urinary culture and sensitivity results and clinical outcome in 25 dogs with UTI	N	CC, BC
Sinha	1980	India	D	11	To establish the correlation between the quantity of formed elements in the urine and the symptoms in determining the course and prognosis of urinary tract infections in 25 dogs	N	None
Siu	2022	USA	C	23	To determine the association between bacterial growth in urine, urinalysis findings and the presence of LUTS in cats with AKI	Y	None
Srivastava	2012	India	D	6*	To compare renal cortical function in healthy and renal failure dogs and detect renal scarring associated with pyelonephritis	N	None
Temizsoylu	2006	Turkey	D	9	To describe the complementary use of radiography and ultrasonography in the diagnosis of upper urinary tract disorders in dogs	N	None
Tripathi	2010	India	D	4	To describe the use of ultrasonography for diagnosis of kidney diseases in dogs	N	None
Vaden	1997	USA	D	2	To examine demographic and clinicopathologic factors that may be associated with the diagnosis and outcome of acute renal failure in dogs presenting to a large referral hospital	N	None
Vrijsen	2021	Belgium	C	5	To document the complications and survival after subcutaneous ureteral bypass placement and to identify risk factors for complications and mortality prior to discharge	N	None
Weese	2022	Canada	C	491	To evaluate initial antimicrobial therapy in cats diagnosed with upper or lower bacterial urinary tract infections at a subset of veterinary practices in the USA and Canada	N	None
Weese	2021	Canada	D	326	To evaluate initial antimicrobial therapy in dogs diagnosed with upper or lower urinary tract infections	N	None
Whitehouse	2024	USA	Cats	32	To describe a clinical population of cats diagnosed with suspected acute PN, including clinical signs, clinicopathological data, imaging findings, targeted bacterial urinary pathogens cultured and microbial susceptibility results, antimicrobial therapy prescribed, and outcomes. To evaluate 3-month survival after hospital discharge. To compare potential clinical markers of disease severity (clinical signs, Haematocrit, white blood cell count, blood urea nitrogen, creatinine, phosphorus, electrolytes, albumin, urine specific gravity, IRIS stage, and urinary tract ultrasound findings) between cats that survived versus non-survivors	Y	Survival to discharge, 1 & 3 months
Wong	2015	USA	D	51	To identify common urinary isolates involved in uncomplicated UTI, complicated UTI and pyelonephritis, as well as their associated susceptibility patterns. To evaluate potential changes in susceptibility patterns over time. To determine whether historical antimicrobial use was associated with future antimicrobial resistance in dogs with UTIs	N	None
Worwag	2008	USA	C	10	To better characterise the clinicopathologic factors and outcome of cats with acute renal failure from a variety of causes	N	None

^1^Diagnostic criteria for PN stated in detail in the study materials and methods or results sections

^2^Clinical outcomes reported specifically for dogs or cats with PN. *Sp* Species, *D* Dogs, *C* Cats, *PN* Pyelonephritis, *CC* Clinical cure, *BC* Bacteriological cure, *HD* Haemodialysis, *UTI* Urinary tract infection, *AKI* Acute kidney injury, *CKD* Chronic kidney disease, *AoC* Acute on chronic, *UO* Urinary obstruction, *SB* Subclinical bacteriuria, *SH* Systemic hypertension, *SAA* Serum amyloid A, *MODS* Multiple organ dysfunction syndrome, *IRIS* International renal interest society

**Table 2:** Study characteristics table for observational studies of pyonephrosis and histologically confirmed pyelonephritis in dogs and cats

Author	Year	Country	Sp	n (PN)	Specific objective(s) of the study
Histologically diagnosed PN					
Bouillion	2018	Canada	D	47	To evaluate the prevalence of PN diagnosed in dogs in an academic referral population, describe the clinical signs and the diagnostic test results in dogs with PN and identify concurrent disorders to determine potential risk factors for PN
Christie	1973	Australia	D	21	To investigate the relationship of primary reflux and pyelonephritis in dogs
Crowell	1975	USA	D	29	To determine the frequency of pyelitis, vasculitis, and renal infarction
DiBartola	1987	USA	C	7	To characterise the historic, physical, laboratory, and histologic findings in cats with chronic renal disease
Goyal	2007	India	D	10 (kidneys)	To describe histopathological findings in 234 canine kidney specimens with macroscopic abnormalities
Kolbjornsen	2008	Norway	D	7	To describe renal lesions observed in young Norwegian Boxer dogs with end-stage renal disease
Lucke	1968	UK	D	28	To describe the macroscopic and histological appearances of 93 examples of renal disease in the domestic cat, together with bacteriological and serological findings. To describe the effects of renal disease on the cardiovascular system, parathyroid glands and bones
Minkus	1994	Germany	D/C	1 (cat)	To compare renal biopsies in cats and dogs with clinical data
Müller Peddinghaus	1977	Germany	D	18 PN, 28 pyelitis	To describe clinical, bacteriological and histopathological findings in dogs without clinical signs of kidney disease
Müller Peddinghaus	1977	Germany	D	35	To investigate if urine polyacrylamide electrophoresis can be used to differentiate tubular from glomerular proteinuria
Müller Peddinghaus	1976	Germany	D	47	To describe the aetiology of glomerulonephritis and chronic interstitial nephritis
Nash	1994	Australia	D	2	To summarise clinical and pathological observations on 7 young SCWT dogs that were investigated for renal disease
Oghiso	1982	Japan	D	4	To compare the results of histopathological examinations of autopsied beagles with published findings
Pachaly	1967	Germany	D	26	To describe histological, pathophysiological, and bacteriological findings in dogs with no obvious signs of kidney disease
Schwarz	1991	Germany	D	2	To investigate E.*coli* isolates and histopathology from the uterus and urinary tract in dogs
Renk	1971	Germany	D	42	To describe the importance of prostatic and uteric disease for the development of urologic and renal disease
Riedel	1969	Germany	C	15	To describe histopathological findings in the urinary tract of 221 autopsied cats
Wettimuny	1963	UK	D	13	To histopathologically describe different types of nephritis in the dog
Pyonephrosis					
Choi	2010	Korea	D	18	To characterise the comparative clinical and ultrasonographic features of pyonephrosis vs. hydronephrosis in dogs
Kuntz	2015	USA	D	13	To describe the technical and clinical outcome of retrograde endoscopic- or fluoroscopic-guided ureteropelvic lavage and ureteral stent placement as a renal-sparing option for the treatment of obstructive pyonephrosis in dogs
Manoharan	2024	USA	D	4	To describe cases where medical dissolution was utilised for the treatment of upper urinary tract nephroureterolithiasis caused by suspected struvite uroliths
Merindol	2023	Canada	C	11	To describe the clinicopathologic findings and outcome in cats with benign urinary tract obstruction that underwent medical management for at least 72 h and compare these findings to those in cats that underwent SUB placement or were euthanised within the first 72 h after presentation. To determine if any pretreatment features predicted success or failure of medical management

*Sp* Species, *D* Dogs, *C* Cats, *PN* Pyelonephritis

**Table 3:** Study characteristics table for case reports of pyelonephritis in dogs and cats

Author	Year	Country	Sp	n	Comorbidities	Urinary pathogen	Antimicrobial treatment	NE	Outcome PN
Abraham	2003	Australia	D	1	Renal dysplasia	E *coli*	None	N	NA
Ackerman	1980	USA	D	5	None	Proteus *mirabilis*, Mycoplasma spp., E *coli*	NR	N	CC&BC (1), CC(1), E(2),Unknown(1)
Agut	2004	Spain	D	1	Renal and cystic uroliths, perinephric abscess	NR	Amoxicillin/clavulanic acid 12.5 mg/kg q12h	Y	NA
Apparicio	2007	Brazil	D	1	Uterine stump pyometra	NR	metronidazole 15 mg/kg q12h, 7 days and amoxicillin 22 mg/kg q12h, 14 days	Y	CC
Arenas	2017	UK	D	1	Nephroliths, ureterolith	Staphylococcus spp., Streptococcus spp.	Amoxicillin, marbofloxacin	N	CC
Arora	1973	India	D	1	BPH	Gram-positive cocci	None	N	NA (post mortem)
Boo	2022	Australia	C	1	Endophtalmitis	E *coli*	Cephazolin 22 mg/kg q8 3 days, amoxicillin/clavulanic acid 14 mg/kg q12h > 17 days	N	D
Bovee	1971	USA	D	1	Urolithiasis, chronic cystitis, chronic prostatitis	Proteus spp.	Furadantin, amoxicillin	N	D
Bovee	1989	USA	D	1	Hypertension	E *coli*	NR	N	NR
Brandt	1971	USA	C	1	NR	NR	Penicillin G injection once	Y	CC
Breitschwerdt	1981	USA	D	1	Nephrogenic DI	E *coli*	ampicillin 23 mg/kg q8h, 10 days then tetracycline 23 mg/kg q8h, 10 days, then TMS 22 mg/kg q12h,10 days	N	CC
Buckley	2019	USA	D	1	DM, nephroliths, emphysematous PN	E. *coli*, "other Gram-negative rods"	Enrofloxacin 9.1 mg/kg q24h and amoxicillin/clavulonic acid 10 mg/kg q12h	N	CC
Buduk	2017	India	D	1	Ureteral calculi, pyonephrosis	E. *coli*	None	N	D
Carter	1976	UK	D	1	DM	NR	NR	Y	E
Choi	2012	Korea	D	1	NR	NR	NR	Y	NR
Cray	2018	USA	C	4	uroliths (3), ureteral stricture (1)	E. *coli* (2) "cocci" (1) Enterococcus spp. (1)	Ampicillin 22 mg/kg q8h, 3 days (changed to amoxicillin/clavulanic acid 14 mg/kg q12h, 14 days) and enrofloxacin 5 mg/kg q24h, 20 days; enrofloxacin 5 mg/kg q24h, 50 days and amoxicillin/clavulanic acid 14 mg/kg q12h, 50 days (and metronidazole for diarrhoea); marbofloxacin 5 mg/kg q24h, 36 days and amoxicillin/clavulanic acid 13 mg/kg q12h, 36 days; marbofloxacin 8 mg/kg q24h and chloramphenicol 25 mg/kg q12 h), then amoxicillin 25 mg/kg q12h and marbofloxacin 9 mg/ml q24h. Treatment length unknown	N	CC/BC; CC; CC/BC; E
Crow	1976	USA	D	1	(Previous automobile accident)	S. *typhimurium*	Chloramphenicol 100 mg/kg/d 17 days (and surgery)	Y	CC
Cupak	1984	Germany	D	1	(Previous gunshot accident)	NR	NR	Y	CC
de Brito Galvao	2013	USA	D	1	Hyperadrenocorticism, nephroliths, suspected urinary bladder malignancy	E. *coli*	Enrofloxacin 12 mg/kg q24h, 56 days	N	CC/BC
Drazner	1980	USA	C	1	NR	E. *coli*	Cephalosporins (IV and PO)	N	CC
Dupont	2015	Belgium	D	1	Suspected CKD	E. *coli*	Amoxicillin/clavulanic acid 12,5 mg/kg q12h, 52 days	N	CC
Fabbi	2016	Italy	D	1	DM, emphysematous cystitis	E. *coli*	Enrofloxacin 5 mg/kg q24	N	D
Fleischhacker	2014	Australia	D	1	Ectopic ureters (and previous 3 months ab)	C. *urealyticum*	Linezolid 30 mg/kg q12h	N	CC (with relapse)
Fuller	1976	USA	D	1	NR	E. *coli*	Amoxicillin 20 mg/kg q6h	Y	CC
Gould	2016	USA	C	1	NR	E. *coli*	Enrofloxacin 5 mg/kg IV q24h and ampicillin 30 mg/kg IV q8h, enrofloxacin 5.6 mg/kg q24h, unknown duration	N	CC/BC
Granito	2015	Canada	D	1	NR	E. *coli*	Enrofloxacin and ampicillin	Y	D
Gupta	2021	India	D	1	NR	NR	Enrofloxacin 5 mg/kg q24h, 6 days	Y	CC
Gupta	2024	India	D	1	End-stage CKD	E. *coli*	Norfloxacin 5 mg/kg q12h	N	D
Harvey	1985	USA	D	1	Necrotizing prostatitis, endocarditis	Serratia *rubidae*	Chloramphenicol 44 mg/kg q6h	N	E
Heathcote	2024	UK	D	1	MCT, uretero-jejunal fistula	E. *coli*	Amoxicillin/clavulanic acid 20 mg/kg q12h, 15 days, marbofloxacin 2.5 mg/kg q24h, 14 days	N	E
Holmes	2019	Australia	D	1	None identified	E. *coli*	Amoxicillin/clavulanic acid	N	CC/BC
Holzworth	1954	USA	C	1	T gondii infection	NR (histopathologic diagnosis)	Chloramphenicol (for Toxo)	N	E
Hotz	1990	USA	C	1	Cardiomyopathy	NR	None (histopathological diagnosis)	N	E
Jamieson	2001	UK	D	1	Renal glucosuria	NR (on antibacterial treatment)	Amoxicillin/clavulanic acid 12.5 mg/kg q12h, 27 days	N	CC/BC
Jergens	1988	USA	D	1	Suspected trigone obstructive neoplasia	E. *coli*	Trimetoprim/sulphadiazine 15 mg/kg q12h, 42 days	N	E
Johnston	1989	UK	D	1	Multiple urinary malformations	P. *mirabilis*, E. *coli*, Streptococcus spp.	Amoxicillin once	N	E
Jones	2018	USA	D	1	Urinary bladder exstrophy, recurrent UTIs	P. *vulgaris*, P. *aeruginosa*	Enrofloxacin 20 mg/kg q24h, and meropenem 12.5 mg/kg q24h	N	E
Kar	2024	India	D	1	NR	E. *coli*, Staphylococcus spp.	Enrofloxacin 19 days	N	CC
Kessler	2008	Germany	D	1	Polycythemia	Klebsiella spp.	Enrofloxacin 5 mg/kg q24h, 10 days	Y	CC/BC
Kim	2020	Korea	D	1	NR	E. *coli*	Enrofloxacin 4 days	N	CC
Kim	2023	Korea	C	1	NR	E. *coli*	Cefotaxime 10 mg/kg q12h and metronidazole 10 mg/kg q12h (and nephrectomy)	Y	CC
Klausner	1980	USA	D	1	Cystic calculus	S. aureus	NR (died 2 h after surgery)	N	D
Kleine	1964	USA	C	1	Recent pyometra	E. *coli*	Oxytetracycline	Y	CC
Kwak	2020	Korea	D	1	Calcium oxalate uroliths	E. *coli*	Amoxicillin 22 mg/kg q8h and enrofloxacin 5 mg/kg q24h, unknown duration	N	CC
Kyles	1996	USA	D	1	NR	S. *intermedius*	Cefalexin 22 mg/kg 14 days	Y	CC
Langner	2016	Germany	C	1	DM	E. *coli*	Amoxicillin/clavulanic acid 12.5 mg/kg q12h, 5 days, marbofloxacin 2 mg/kg q24h, 28 days	N	CC/BC
Lee	2010	Korea	C	1	Renal subcapsular abscess	"rod-shaped bacteria"	Enrofloxacin 2.5 mg/kg q12h and metronidazole 15 mg/kg q12h, 10 days, meropenem	Y	CC
Lettow	1974	Germany	D	2	Ectopic ureters	P. vulgaris (1) NR (1)	NR	Y	NR
Lewis	1988	Australia	D	1	Nephrolith, perirenal abscess	Staphylococcus spp.	Amoxicillin 100 mg q12h, 12 days	Y	CC
Lieske	2021	USA	C	1	Gastrointestinal smooth muscle alpha-actin deficiency	"cocci"	NR (post-mortem diagnosis)	N	NA
Lim	2016	Korea	D	1	EHPSS, ureterolith	E. *coli*	NR	Y	CC
Luntz	1984	USA	C	1	NR	NR	Ampicillin 30 mg	N	D
Madewell	1975	USA	D	1	Cystic calculus	E. *coli*, S. *aureus*	Chloramphenicol 55 mg/kg q6h, 7 days, nitrofurantoin 4.4 mg/kg q8h, 7 days, sulfisoxazole 44 mg/kg q3h, duration unknown	N	CC
Magid	1994	USA	D	1	Suspected HAC, suspected nephrolithephro	P. *mirabilis*	Amoxicillin/clavulanic acid 15 mg/kg q12h, 5 days, cephalexin 15 mg/kg q12h	N	E
Maier	2014	Germany	D	1	Ureteroliths	NR (on antibacterial treatment)	Amoxicillin/clavulanic acid 10 mg/kg q12h, 7 days	Y	CC
Montasell	2020	Canada	D	1	Ureteroliths, uretero-cutaneous fistula	Staphylococcus spp.	Enrofloxacin 9 mg/kg q24h, 42 days and amoxicillin/clavulanic acid 14 mg/kg q12h, 42 days	N	CC/BC (with relapse)
Moon	2014	USA	D C	2	DM (cat) Suspected HAC (dog)	E. *coli* and Enterobacter *cloacae* (dog). E. *coli* and Streptococcus spp. (cat)	Enrofloxacin 5.8 mg/kg q12h and ampicillin 28 mg/kg q8h, imipenem 8 mg/kg q8h (dog), ampicillin 33 mg/kg q3h, 5 days, amoxicillin/clavulanic acid 20 mg/kg q12h, duration unknown	N	D (dog), E (cat)
Muhammad	2017	Nigeria	D	1	urethral defect	Corynebacterium spp., Staphylococcus spp.	Intracystic enrofloxacin and streptomycin 30 min, 5 days and enrofloxacin 5 mg/kg q24h, 7 days	N	CC
Müller Peddinghaus	1978	Germany	D	1	Gunshot wound	S. *aureus*	NR (post-mortem diagnosis)	N	E
Oh	2017	Korea	D	2	Nephro-cutaneous fistula, nephroliths (1), ureteral obstruction (1)	S. *aureus* (1), E. *coli* (1)	NR	Y	CC (2)
Peli	2003	Italy	D	1	DM and HAC (1), DM (1)	E. *coli* (2)	NR	N	NR
Phelps	2022	USA	D	1	NR	S. *canis*, relapse: E *coli*	NR (initial visit) enrofloxacin 14 days (relapse)	N	NR
Phipps	2020	UK	D	1	Circumcaval ureter	S. *pseudo-intermedius*	Cefuroxime 20 mg/kg q8h, 1 day, enrofloxacin 5 mg/kg q24h, unknown duration	Y	NR
Pointer	2011	USA	C	1	Chronic prostatitis, balanoposthitis	NR	None	N	E
Pozzi	2006	USA	D	1	Neoureterocystotomy	Enterococcus spp.	Amoxicillin/clavulanic acid	N	CC/BC
Read	1978	Australia	D	1	Lower urinary tract obstruction	NR	NR (post-mortem diagnosis)	N	E
Robin	2019	France	C	1	Multiple urinary malformations	E. *coli*	Marbofloxacin 4 mg/kg q24h	N	E
Sakatani	2018	Japan	C	1	Systemic amyloidosis	NR	Enrofloxacin 5 mg/kg q24h, unknown duration	N	D
Sarkar	1979	India	C	1	NR	C. *pyogenes*	NR	N	D
Schaer	1992	USA	C	1	Urethral stenosis	S. *aureus*	Cefalexin 100 mg q12h, 14 days	N	CC
Speake	1989	Denmark	D	1	NR	E. *coli*	NR	Y	E
Szatmari	2001	Hungary	D	2	NR	S. *aureus* (1) NR (1)	Amoxicillin/clavulanic acid 20 mg/kg q12h and metronidazole 15 mg/kg q12h until C&S, thereafter enrofloxacin 5 mg/kg q24h, 35 days (1), amoxicillin/clavulanic acid 12.5 mg/kg q12h, unknown duration	N	CC/BC (2)
Thiet	1991	Germany	D	1	ureteral obstruction	Citrobacter spp., Streptococcus spp.	Penicillin G 20000 IE/kg q24h and gentamicin 3 mg/kg q12h, then enrofloxacin 5 mg/kg q24h, unknown duration	N	CC
Vedrine	2017	France	C	1	Ureteral obstruction, CKD	E. *coli*	Cefovecin injections day 1 and 15	N	CC
Veni	2018	India	D	1	CKD	E. *coli*	None	N	E
Vernier	2024	USA	C	2	None (1), ureteroliths (1)	Streptococcus spp. (1), NR (1)	Amoxicillin/clavulanic acid 16.6 mg/kg q12h, unknown duration (1), NR (1)	N	CC (1), E (1)
Watson	1993	USA	C	1	Discospondylitis, CKD	E. *coli*	Long-term ampicillin	N	E
Watson	1986	Australia	C	1	NR	E. *coli*	Amoxicillin 75 mg q12h 18 days	N	CC
Williams	2005	South Africa	D	1	Lymphangiosarcoma, CKD	NR	NR (post-mortem diagnosis)	N	E
Wilson	2024	Australia	C	1	Myositis	S. *typhimurium*	Amoxicillin/clavulanic acid 20 mg/kg q12h 14 days	N	CC/BC
Zatelli	2014	Italy	C	1	NR	E. *coli*	Enrofloxacin 5 mg/kg q24h	N	D

*Sp* Species, *D* Dogs, *C* Cats, *PN* Pyelonephritis, *NR* Not recorded, *NE* Nephrectomy yes/no, *CKD* chronic kidney disease, *CC* Clinical cure, *BC* Bacteriological cure, *E* Euthanasia

### Diagnostic criteria and reported outcomes

**Fig. 3:**
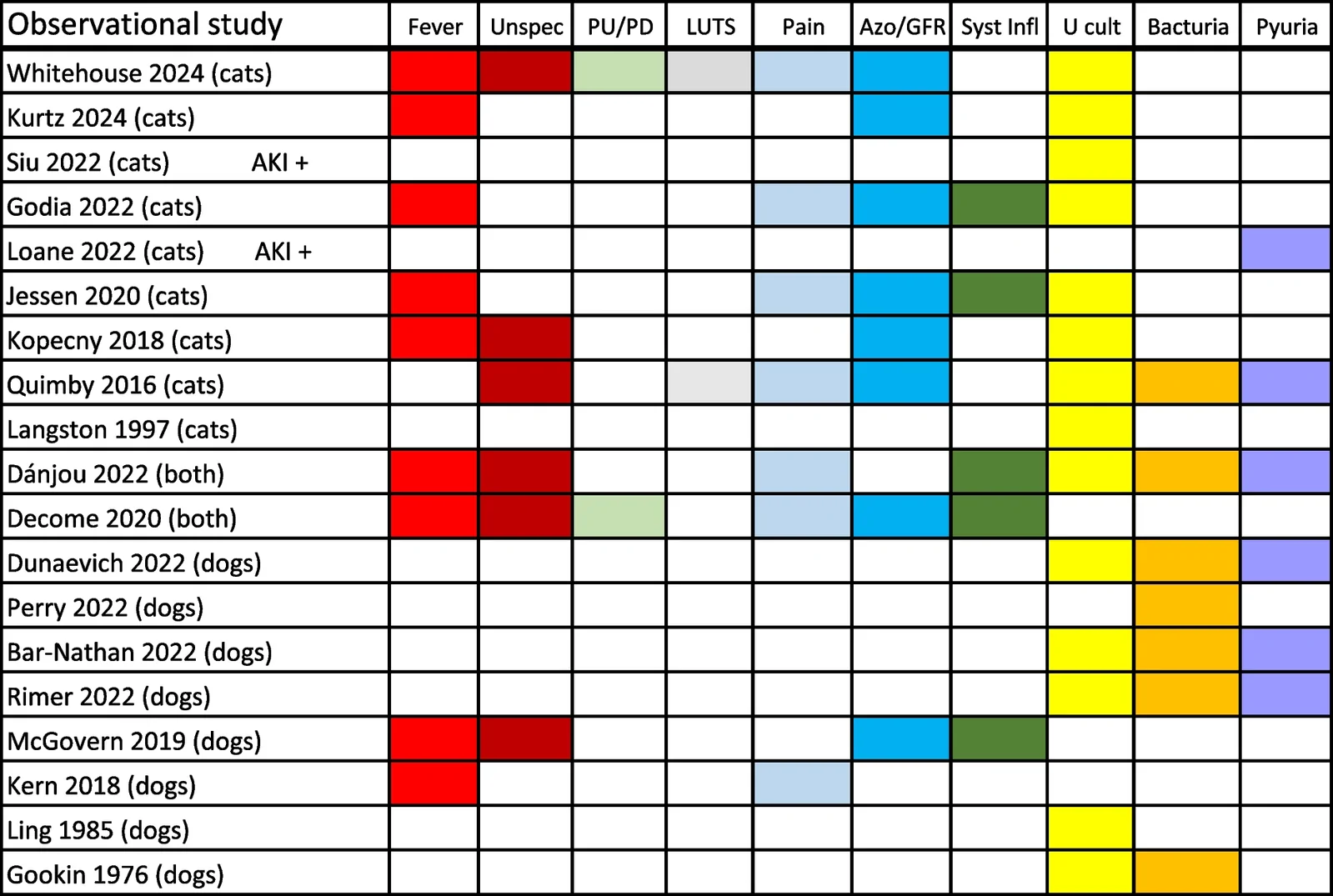
Overview of diagnostic criteria used for the diagnosis of PN. Unspec: unspecific signs such as lethargy, inappetence, or anorexia. PU/PD: polyuria and polydipsia. LUTS: lower urinary tract signs. Pain: abdominal or renal pain. Azo/GFR: azotaemia or another sign of decreased GFR. Syst infl: systemic inflammation. U cult: urinary culture

Clinical diagnostic criteria for the diagnosis of PN were provided in only 19 studies. These criteria were pre-defined in the methods section in 17 of these 19 studies and presented in the results section in two studies. The specific clinical diagnostic criteria used for diagnosis of PN are provided in Table 4. The criteria were heterogeneous but typically combined elements from two or more of four domains, microbiology (most required a positive urine culture of urine obtained by cystocentesis, with a minority using pyelocentesis as a reference standard, or bacteriuria and pyuria on sediment instead of culture), clinical signs (most commonly lethargy, inappetence, vomiting, fever, and renal or abdominal pain), clinicopathology (azotaemia, leucocytosis, left shift, or increased acute phase proteins), and imaging (renal pelvic dilation, proximal ureteral dilation, hyperechoic cortices, retroperitoneal changes). In Fig. 3, a summarizing overview of diagnostic criteria used for the diagnosis of PN is presented.

**Table 4:** Clinical diagnostic criteria for pyelonephritis in dogs and cats in the 19 studies that provided such data

Author	Year	Sp	Pre-def^1^	Clinical definition of pyelonephritis
Whitehouse	2024	C	YES	Cats w a clinical diagnosis of suspected acute PN were include IF met All 4 of the following criteria: (1) confirmed bacteriuria by a positive aerobic bacterial urine culture collected via cystocentesis; (2) presence of at least 1 consistent clinical sign of AKI such as lethargy, hyporexia, anorexia, vomiting, polyuria/polydipsia, periuria, haematuria, fever, and renal or abdominal pain; (3) acute clinical signs that developed within 1 week of presentation; and (4) new or worsening azotaemia (increase in creatinine of ≥ 0.5 mg/dL since the last documented value) as defined by the IRIS as an AKI grade II or higher
Dunaevich	2022	D	YES	Positive urine culture, urine sediment findings (pyuria, bacteriuria), and ultrasonographic findings consistent with pyelonephritis
Kern	2018	D	YES	Presence of fever, abdominal pain, ultrasonographic changes consistent with pyelonephritis (eg, perinephric free fluid, pyelectasia, hyperechoic renal cortices, or some combination of these), or based on the diagnosis of the attending clinician
Decome	2022	D	YES	At least one of the following: elevated body temperature (rectal temperature > 39.4 °C), vomiting, lethargy, loss of appetite, pain elicited by renal palpation, or polyuria/polydipsia. AND Renal azotaemia ± abnormal leucocyte count or toxogram AND ultrasonographic evidence of pyelonephritis (e.g., renal pelvis dilation, retroperitoneal effusion, perineal steatitis, increased echogenicity of the renal parenchyma) AND improvement of azo or imaging findings after ab
Perry	2022	D	YES	Renal pelvic and ureteral dilation, a hyperechoic renal cortex and retroperitoneal space, and poor corticomedullary definition on ultrasound in conjunction with bacteriuria on urinalysis obtained via cystocentesis
Bar-Nathan	2022	D	YES	Based on urine sediment findings (pyuria and bacteriuria), positive urine culture, and ultrasonographic findings
Godia	2022	C	YES	Either a positive pelvic urine culture or a clinical presentation highly suggestive of PN, defined as presence of pyrexia, renal pain, azotaemia and/or elevated acute phase proteins, along with a positive bladder urine culture. Cats fulfilling those criteria were classified as PN regardless of whether additional urological comorbidities were present or not
D’Anjou	2022	D C	YES	Combination of positive urine culture and/or presence of bacteria and increased white blood cell count on cystocentesis or pyelocentesis, an inflammatory leucogram, and relevant clinical signs such as fever, lethargy, and renal pain
Loane	2020	C	YES	In addition to AKI or CKD criteria: pyelectasia, active urine sediment, clinical and clinicopathological improvement after antibiotic administration. ± positive culture (not required)
Jessen	2020	C	YES	Positive urine culture obtained by pyelocentesis (pelvic urine culture) or cats with a presentation highly suggestive of PN were classified as PN regardless of whether urological comorbidities were present or not. A clinical presentation highly suggestive of PN was defined as presence of pyrexia, renal pain, azotaemia, elevated acute phase proteins combined with a positive cystocentesis urine culture, and resolution of clinical signs and laboratory abnormalities in response to antimicrobial treatment
Kopecny	2018	C	YES	Positive urine culture was present in combination with a progressive increase in serum creatinine concentration from baseline, the presence of systemic clinical signs (ie, lethargy, hyporexia, vomiting, and fever), or both. UTI supported by resolution of clinical signs after ab
Ling	1985	D	YES	Unilateral or bilateral culture-positive renal pelvic urine in addition to positive bladder culture
Rimer	2022	D	YES	Positive urine culture, urine sediment findings (pyuria and bacteriuria), and ultrasonographic findings consistent with pyelonephritis, as described in Wettimuny 67
McGovern	2019	D	YES	Pyelonephritis was diagnosed by clinicians on the basis of clinical findings (unexplained anorexia, lethargy, and/or fever), haematology (leucocytosis), serum chemistry (azotaemia), and abdominal imaging (pyelectasia, dilation of the proximal ureter, abnormal appearance of the kidneys)
Quimby	2016	C	YES	Cats with a clinical diagnosis of pyelonephritis were defined as those undergoing diagnostics and treatment for uremic crisis (elevation in serum creatinine from previous baseline) with bacteriuria on urinalysis collected via cystocentesis and positive urine culture, or an active sediment suspicious of UTI on urinalysis collected via cystocentesis. Concurrent ureterolith(s) was an exclusion criterion for this group. These cats also had to have two or more history and physical examination parameters consistent with pyelonephritis, including anorexia or inappetence, lethargy, vomiting, periuria, haematuria, weight loss, or renal or abdominal pain. Presence of pyelectasia on ultrasound was not used as an inclusion criterion. Final determination of clinical diagnosis of pyelonephritis was made after response to therapy was assessed and repeat ultrasound was performed to confirm improvement of dilation of pelvis and/or ureters
Kurtz	2024	C	YES	Confirmation (1a) or exclusion (2a) of bacterial pyelonephritis was based on a positive or negative bacterial culture on urine sampled via ultrasound-guided pyelocentesis. Presumptive diagnosis of bacterial pyelonephritis (1b) was based on compatible history and clinical evolution (assessed by a consensus among 3 board-certified internal medicine specialists as well as at least 3 of the following criteria: azotaemia (defined as serum creatinine concentration above the upper limit of the reference interval), positive bacterial culture on urine collected by cystocentesis or from a SUB access port, hyperthermia (rectal temperature ≥ 39.5_C), presence of renal pelvic dilation (≥ 1 mm on transverse ultrasonographic view). Bacterial pyelonephritis was judged unlikely (2b) based on history and clinical evaluation and if < 3 of the above criteria were met. Cats on antimicrobial treatment and negative cultures on pyelocentesis were also included in Group 2b, provided they fulfilled the aforementioned criteria
Siu	2022	C	YES	In cats with AKI, pyelonephritis was suspected based on a positive urine culture or confirmed based on culture of urine collected by pyelocentesis
Langston	1997	C	NO	Ultrasonographic findings of dilated renal pelves, positive urine culture, or response to antimicrobial treatment
Gookin	1976	D	NO	Two or more of the following conditions were present: chronic or resistant urinary tract infection, unilateral renal abnormalities consistent with pyelonephritis on excretory urograms or abdominal ultrasonograms, poor renal function in the affected kidney, as determined by means of renal scintigraphy, and infection confirmed to be limited to 1 kidney by means of Gram's staining or aerobic bacterial culturing of urine samples collected from the ureters

*Sp* Species, *D* Dogs, *C* Cats, *PN* Pyelonephritis, *Pre-def* Clinical diagnostic criteria for PN were pre-defined in the methods section of the study

Only a few observational studies reported clinical outcomes, most commonly short-term survival. Long-term renal functional data in dogs or cats with PN are virtually absent (Table 1). Common urinary comorbidities described in dogs and cats with PN included urolithiasis (cystic, renal and ureteral), chronic kidney disease and congenital abnormalities such as ectopic ureters. A few observational studies reported descriptive proportional data regarding comorbidities within or outside the urinary tract, infecting agents, clinical signs and laboratory or ultrasound abnormalities. Data from these studies are summarised in Table 5.

**Table 5:** Extraction of proportional clinical data from studies describing ≥ 10 cases of pyelonephritis in cats or dogs

Clinical data	Included studies (n)	Cats (pooled proportions)	Dogs (pooled proportions)	Comments
Comorbid uropathies in cats and dogs with PN	Cats (n = 3): [22, 101, 183]	Chronic kidney disease: 52% (22/42) Uroliths (upper and lower) 46% (27/59)	Chronic kidney disease: 19% (3/16) Uroliths (upper and lower) 37% (6/16) Recurrent UTIs 69% (11/16) Anatomic abnormality 12% (2/16)	AKI is not listed because it is a common sequel of PN
	Dogs (*n* = 1): [141]			
Comorbid conditions outside the urinary tract in cats and dogs with PN	Cats (*n* = 1): [183]	Hyperthyrodism: 19% (6/32) Cardiomyopathy: 9% (3/32) Pancreatitis: 3% (1/32) Diabetes mellitus: 6% (2/32) Feline asthma 6% (2/32) Chronic enteropathy 6% (2/32)	Hyperadrenocorticism 12% (2/17) Thoracolumbar myelopathy 12% (2/17) Costicosteroid administration 24% (4/17)	
	Dogs (*n* = 1): [141]			
Pathogens causing PN in cats and dogs	Cats (*n* = 3): [22, 101, 183]	E *coli*: 82% (53/65) Pseudomonas spp. 3% (2/65) Staphylococcus spp. 5% (3/65) Proteus spp. 3% (2/65) Streptococcus spp. 2% (1/65) Enterococcus spp. 6% (4/65)	E *coli*: 54% (70/129) Pseudomonas spp. 4% (5/129) Staphylococcus spp. 10% (13/129) Proteus spp. 7% (9/129) Streptococcus spp. 2% (2/129) Klebsiella spp. 6% (8/129) Enterobacter spp. 5% (7/129) Enterococcus spp. 6% (8/129)	In most studies the number of isolates were higher than the number of dogs with PN. The number of isolates are used for pooled calculations
	Dogs (*n* = 4): [75, 124, 164, 186]			
Clinical signs in cats and dogs with PN	Cats (*n* = 2): [101, 183]	Anorexia: 81% (40/49) Lethargy: 59% (29/49) Abdominal pain: 37% (12/32) Vomiting: 35% (17/49) Fever: 22% (7/32) PU/PD: 16% (8/49) Stranguria: 14% (7/49) Haematuria: 12% (6/49) Pollakiuria: 12% (6/49)	NR	Anorexia, lethargy and vomiting were part of the diagnostic criteria for PN in a number of studies, possibly creating a bias towards these clinical signs for the proposed proportion of animals with these criteria
	Dogs (*n* = 0)			
Haematological and biochemical abnormalities in blood from cats and dogs with PN	Cats (n = 2): [101, 183]	Anemia: 61% (17/28) Neutrophilia: 63% (24/38) Left shift: 50% (14/28) Monocytosis: 30% (3/10) Azotaemia: 98% (48/49) Hyperglobulinemia: 100% (7/7)	NR	AKI and/or azotaemia was part of study inclusion or diagnostic criteria for many studies, creating a strong bias towards increased creatinine for the proposed proportion of animals with these criteria
	Dogs (*n* = 0)			
Ultrasonographic abnormalities in cats and dogs with PN	Cats (*n* = 3). [101, 147, 183]	Pyelectasia: 80% (40/50) Ureteral dilatation: 64% (32/50) Renal hyperechogenicity: 40% (15/37) Retroperitoneal hyperechogenicity: 37% (14/37) CKD (small, irregular kidneys): 50% (10/20) Renomegaly: 15% (3/20)	NR	Renal pelvic dilation was part of the diagnostic criteria for many studies, creating a strong bias towards pelvic dilation for the proposed proportion of animals with this criterion
	Dogs (*n* = 0)			

### Diagnostic test accuracy studies (biomarkers and imaging)

Six studies evaluated diagnostic tests for PN. Two feline studies assessed serum amyloid A (SAA) with comparator groups (Kurtz et al*.*, Degenhardt et al.). Kurtz et al. (2024) evaluated SAA in cats with suspected/confirmed bacterial PN versus cats with lower urinary tract disease or chronic kidney disease (CKD) and concluded that an SAA > 51.3 mg/L was suggestive of PN. In this study, cats with ureteral disease, including ureteral obstruction, were excluded. Degenhardt et al. compared SAA among groups of cats with AKI of different aetiologies, azotaemic CKD, and acute-on-chronic kidney injury. Serum amyloid A could not discriminate among groups but 4/4 cats with azotaemic PN had an SAA above 100 mg/L. The remaining one cat with a diagnosis of PN in this study was not azotaemic and had an SAA within the reference interval.

Two studies explored urinary microRNA (miRNA) profiles as potential discriminatory markers for PN versus other urological diseases. Jessen et al. suggested that upregulation of miRNA16 might be PN-specific, pathogen-specific (*Escherichia coli*), or both. Godia et al*.* found 7 miRNAs that were differentially abundant between control and PN cats, and another 6 miRNAs that were relevant for discriminating PN from other urological conditions (including ureteral obstruction).

Renal imaging studies with comparator groups were limited to two [2, 147]. Renal pelvic width measured using ultrasonography in dogs and cats with normal renal function (with and without diuresis) were compared to pelvic width in animals with nonobstructive urinary tract disease (AKI, CKD, PN, ectopic ureter and urinary tract neoplasia) and partial to completely obstructive urinary tract disease [2]. A substantial overlap in pelvic width across groups was shown. In cats, Quimby et al*.* compared ultrasonographic features among groups of cats with stable CKD, clinically diagnosed PN, and ureteral obstruction, and similarly found considerable overlap in renal pelvic and proximal ureteral dilation among groups, indicating that these measurements alone are insufficient to distinguish PN from ureteral obstruction or CKD [147]. Taken together, these imaging studies suggest that renal pelvic or proximal ureteral width measured by ultrasound should not be used as a standalone diagnostic test for PN.

### Antibacterial treatment and outcome

No prospective trials were comparing the efficacy among different antibacterial substances or treatment durations. Whitehouse et al. provided the only cohort-level antibacterial treatment data for PN, and in that study, *E*. *coli* comprised 89% of 38 isolates from 32 cats with PN [183]. In 18 cats, a potentiated amoxicillin was used, and in 12 cats, a fluoroquinolone was used. Eight of these 12 cats were given pradofloxacin and 4 were given enrofloxacin. The empirically chosen antibacterial substance was changed when culture results were available in 11 (39%) of cats. Median treatment duration was 29 days (range, 10–62). Among 12 cats with post-therapy cultures, bacteriologic cure occurred in 10 (nine of them had received a fluoroquinolone, one had received cefodoxime). Two cats remained bacteriuric (*E*. *coli*), one of these had received a fluoroquinolone, and 1 had received cefovecin. Survival to discharge was 94% (29/31), and 3-month survival among cats with follow-up was 70% (14/20). Non-survivors had higher admission serum creatinine, BUN, and phosphorus concentrations than survivors [183].

Antibacterial treatment and outcomes were reported in 93 animals across 81 case reports (Table 3). Amoxicillin/clavulanic acid and fluoroquinolones were the most frequently used antibacterials. Enrofloxacin was the most prescribed fluoroquinolone substance, used in both dogs and cats (Table 3). Reported treatment durations typically ranged from 2 to 6 weeks. No reliable comparisons could be made about treatment efficacy among substances because cases varied widely in severity, comorbidities, and reporting detail.

### Histopathology studies and pyonephrosis

Studies describing PN diagnosed by histopathology and pyonephrosis were two distinct themes that stood out among included studies (Table 2). In histopathology studies, necropsy or biopsy series of cases in which PN was diagnosed by tissue diagnosis were described. These reports included both acute suppurative and chronic renal interstitial patterns, frequently together with other urinary tract pathology, such as ectopic ureters. In some of these studies, culture or bacteriologic data implicated Enterobacterales (*E. coli*, *Proteus* spp.) as infecting agents. Because case identification was often post-mortem and clinical information and antibacterial data were limited, some cases are likely to have been subclinical. Nevertheless, these studies present plausible pathophysiologic pathways (ascending infection, reflux, anatomic abnormalities) and suggest that PN can be present without clear ante-mortem localizing clinical signs.

The pyonephrosis studies focused on obstructive, pus-filled renal pelves and emphasised imaging characteristics and interventions. Procedural series described renal-sparing approaches such as retrograde ureteropelvic lavage with ureteral stenting in dogs and subcutaneous ureteral bypass devices in cats. Only short-term clinical resolutions were documented.

## Discussion

In this study, a systematic search of databases was performed to identify published scientific studies of all aspects of PN in dogs and cats. In the 176 studies that were finally included, three distinct entities were identified: histologically diagnosed PN, clinically diagnosed PN and pyonephrosis. Histologically diagnosed and clinically diagnosed PN represent different diagnostic approaches and may therefore identify somewhat different patient populations. Pyonephrosis, accumulation of purulent material in the renal pelvis secondary to an obstruction, could be described as a separate form of PN. Interestingly, pyonephrosis may also develop when accumulated pus secondarily obstructs the ureter in cases of PN. Pyonephrosis is probably usually less difficult to diagnose clinically than unobstructed PN, at least with access to relevant imaging techniques, because of the accumulation of pus in the renal pelvis. Therefore, there should probably be different clinical criteria developed for diagnosis of pyonephrosis compared to nonobstructive PN.

Overall, few studies described clinical data for dogs and cats diagnosed with PN, and when present, clinical diagnostic criteria for this diagnosis varied and were often extensive. This variation leads to different clinical populations in different studies, making evidence synthesis and generalisation of results difficult. In some studies, PN was further characterised as acute or chronic, but often no distinction between acute or chronic was made. The long-term impact of PN on renal health is unclear. While chronic or subclinical PN could lead to tubulointerstitial damage and contribute to CKD development or progression, prospective data linking clinically diagnosed PN to subsequent decline in kidney function are lacking. Conversely, pre-existing CKD may predispose to PN, making causal direction difficult to determine in retrospective cohorts.

In humans, the situation regarding definition of, and diagnostic criteria for, PN seems similarly unclear [190, 191]. The definitions of human UTIs have been proposed to change from uncomplicated or complicated UTI, respectively, to local (cystitis) or systemic (PN or prostatitis with or without sepsis), the latter requiring more intensive diagnostic evaluation [192]. In recent human guidelines, however, the terms uncomplicated UTI and complicated UTI are still used [193, 194].

The feline studies evaluating the diagnostic value of serum SAA and urinary mRNAs for the diagnosis of PN [22, 85] indicate that there is a perceived need for PN-specific biomarkers for diagnosis. In humans, markers of systemic inflammation such as C-reactive protein and procalcitonin can be used to evaluate involvement of the renal parenchyma. In a Cochrane review from 2020, none of these tests were considered accurate enough to allow clinicians to confidently differentiate upper from lower urinary tract disease in children, mainly because of a low specificity [195]. More specific urinary markers of renal involvement (for example, urinary interleukins) have been evaluated in people but are not yet used in clinical practice [196, 197]. Further studies of sensitive and specific biomarkers for the diagnosis of PN seem to be needed in both human and companion animal medicine.

Ultrasound evaluation of the urinary tract in people with PN is performed to rule out urinary tract abnormalities such as voiding dysfunction, reflux, and renal abscesses or masses [193, 197]. In dogs and cats, ultrasound is also used to rule in PN. The specificity of this diagnostic test is not known but suspected to be low. Two studies compared renal pelvis width among cats with different renal conditions and the overlap was large, supporting further evaluations of the specificity of ultrasound for the diagnosis of PN in dogs and cats.

The choice of antibacterial substance varied across studies included in this review, and this is a controversial area in veterinary medicine. Certain antibacterial classes (for example, fluoroquinolones) are considered more appropriate for the treatment of PN in dogs and cats than, for example, beta-lactams [1]. Interestingly, a recent human systematic review and meta-analysis showed that clinical cure of PN was comparable for different classes of antibacterials [198]. The authors concluded that local resistance patterns, patient factors and toxicity profiles should guide antibiotic selection for PN, rather than presumed class superiority. Ultimately, blood concentrations of the chosen substance need to be high enough to achieve adequate levels in the renal parenchyma to eradicate bacteria.

Across the veterinary literature mapped in this scoping review, reported treatment durations for PN in dogs and cats were commonly 2–6 weeks in observational cohorts and case reports, with no prospective trials comparing different durations. Previous veterinary guidelines recommended treatment durations of 4–6 weeks [199], whereas the current recommendation of antibacterial therapy for PN is 10–14 days [1]. However, prescribing data indicate that shorter courses were already used in some settings before the 2019 ISCAID update. In a North American multicentre study of cats with urinary tract infections from 2016–2018 [180], the median duration of treatment for PN was 14 days with no identifiable association between duration and clinic type or country (USA vs. Canada). In Sweden, this recommendation has been adopted in national guidance since 2022, and a multicentre retrospective cohort from Sweden found that most cats and dogs with PN during the years 2022–2024 were treated with antibacterials for ≤ 14 days, indicating guideline compliance (manuscript in preparation). Antibacterial treatment durations for PN have been investigated in several trials in human medicine. Multiple systematic reviews in adults with acute uncomplicated PN indicate that 5–7 days of treatment are as effective as longer courses [200–202]. Guideline recommendations are regimen-specific, with fluoroquinolones typically given for 5–7 days, whereas trimethoprim-sulfamethoxazole or beta-lactams are often prescribed for 10–14 days [194, 203]. In infants and children, 10–14 days of treatment remains common [197, 204]. Taken together, current veterinary recommendation of 10–14 days aligns with paediatric practice and with adult non-fluoroquinolone regimens but exceeds the shortest effective adult fluoroquinolone courses. Randomised studies comparing shorter versus longer durations (for example, 7 vs 14 days) of treatment for PN, stratified by drug class and complicating factors and using standardised outcome measures and follow-up protocols, are a clear research priority in veterinary medicine. Little is also known about optimal monitoring of individuals treated for PN, and there is no consensus regarding this in veterinary medicine. In children who have recovered clinically from an episode of acute PN, no follow-up urine culture is currently recommended because of lack of evidence to support its utility in clinical practice [197].

One objective of this scoping review was to evaluate the feasibility of a subsequent systematic review and, where appropriate, meta-analysis. Mapping identified a few domains with reasonably comparable data to consider quantitative evidence synthesis. Specifically, a prevalence meta-analysis appears feasible for short-term mortality outcomes in PN cohorts and for the proportion of AKI cases attributable to PN within defined AKI populations. In addition, a few diagnostic studies reported extractable data for potential pooling of clinical, biomarker, and imaging indicators.

The study has some limitations. Because of the language restriction, information from studies written in languages other than English or German could not be included in the review. Also, no attempt to reach grey literature was made, and therefore useful information may have been missed.

## Conclusions

Multiple information gaps regarding the clinical aspects of PN in dogs and cats were detected. There are no universally accepted clinical criteria for the diagnosis and no clinical trials evaluating antibacterial (or other) treatment options. Information about short-term outcomes for dogs and cats with PN is scarce, and long-term outcomes do not seem to be investigated at all. Consequently, more research is needed, preferably starting with the development of clinical criteria for diagnosis and core outcomes. Thereafter, controlled clinical trials evaluating different antibacterial treatment options and treatment durations should be performed to improve care of cats and dogs with PN in the future.

## Supplementary Information

Supplementary Material 1.

Supplementary Material 2.

Supplementary Material 3.

## Data Availability

Data sharing is not applicable to this article as no datasets were generated or analysed during this study.
