# Supplementary material for: Pyelonephritis in cats and dogs - a scoping review
Source: BMC Vet Res. 2026 Oct 26;22:635. doi: 10.1186/s12917-026-06042-1 (PMC13712058; doi:10.1186/s12917-026-06042-1)
Supplement: Supplementary file 2 [file 12917_2026_6042_MOESM2_ESM.pdf]

Britt Marie Bergquist and Isabelle Nesterud, SLU library

Initial search, on the 22th of March 2022

**Database**: Biosis<br>**Date**: 2023-03-22		
**Search**	**Terms**	**Result**
1	TS=(cat OR cats OR feline OR Dog OR dogs OR canine OR canis OR felis)	149,040
2	TS=((pyelonephrit* OR nephrit* OR pyelitis OR pyonephros*) OR ((“Acute kidney injur*” OR “Acute kidney failure” OR “Acute Renal failure”) AND (Infect* OR Bacteri*)))	19,277
3	1 AND 2	288

Search query:
TS=(cat OR cats OR feline OR Dog OR dogs OR canine OR canis OR felis) AND TS=((pyelonephrit* OR nephrit* OR pyelitis OR pyonephros*) OR ((“Acute kidney injur*” OR “Acute kidney failure” OR “Acute Renal failure”) AND (Infect* OR Bacteri*)))

**Database**: CABI, Cab Abstracts<br>**Date**: 2023-03-22		
**Search**	**Terms**	**Result**
1	TS=(cat OR cats OR feline OR Dog OR dogs OR canine OR canis OR felis)	384,474
2	TS=((pyelonephrit* OR nephrit* OR pyelitis OR pyonephros*) OR ((“Acute kidney injur*” OR “Acute kidney failure” OR “Acute Renal failure”) AND (Infect* OR Bacteri*)))	14,182
3	1 AND 2	1418

Search query:
TS=(cat OR cats OR feline OR Dog OR dogs OR canine OR canis OR felis) AND TS=((pyelonephrit* OR nephrit* OR pyelitis OR pyonephros*) OR ((“Acute kidney injur*” OR “Acute kidney failure” OR “Acute Renal failure”) AND (Infect* OR Bacteri*)))

**Database**: Medline via WoS<br>**Date**: 2023-03-22		
**Search**	**Terms**	**Result**
1	TS=(cat OR cats OR feline OR Dog OR dogs OR canine OR canis OR felis)	582,583
2	TS=((pyelonephrit* OR nephrit* OR pyelitis OR pyonephros*) OR ((“Acute kidney injur*” OR “Acute kidney failure” OR “Acute Renal failure”) AND (Infect* OR Bacteri*)))	71,010
3	MHX=(Pyelonephritis OR Nephritis, Interstitial OR Pyelitis OR Pyonephrosis OR Acute Kidney Injury)	75,800
4	2 OR 3	118,713
5	1 AND 4	**2,122**

Search query:

TS=(cat OR cats OR feline OR Dog OR dogs OR canine OR canis OR felis) AND (TS=((pyelonephrit* OR nephrit* OR pyelitis OR pyonephros*) OR ((“Acute kidney injur*” OR “Acute kidney failure” OR “Acute Renal failure”) AND (Infect* OR Bacteri*))) OR (MHX=(Pyelonephritis OR Nephritis, Interstitial OR Pyelitis OR Pyonephrosis OR Acute Kidney Injury)))

**Database**: Web of Science, Core collection (SCI-EXPANDED, SSCI, AHCI, ESCI)<br>**Date**: 2023-03-22		
**Search**	**Terms**	**Result**
1	TS=(cat OR cats OR feline OR Dog OR dogs OR canine OR canis OR felis)	590,613
2	TS=((pyelonephrit* OR nephrit* OR pyelitis OR pyonephros*) OR ((“Acute kidney injur*” OR “Acute kidney failure” OR “Acute Renal failure”) AND (Infect* OR Bacteri*)))	57,647
3	1 AND 2	**873**

Search query:
TS=(cat OR cats OR feline OR Dog OR dogs OR canine OR canis OR felis) AND TS=((pyelonephrit* OR nephrit* OR pyelitis OR pyonephros*) OR ((“Acute kidney injur*” OR “Acute kidney failure” OR “Acute Renal failure”) AND (Infect* OR Bacteri*)))

Britt Marie Bergquist and Isabelle Nesterud, SLU library

**Database**: Scopus<br>**Date**: 2023-03-22		
**Search**	**Terms**	**Result**
1	TITLE-ABS-KEY(cat OR cats OR feline OR Dog OR dogs OR canine OR canis OR felis)	768,382
2	(TITLE-ABS-KEY(pyelonephrit* OR nephrit* OR pyelitis OR pyonephros*)) OR ((TITLE-ABS-KEY("Acute kidney injur*" OR "Acute kidney failure" OR "Acute Renal failure")) AND (TITLE-ABS-KEY(Infect* OR Bacteri*)))	117,641
3	1 AND 2	**1,742**

Search query:

(TITLE-ABS-KEY(cat OR cats OR feline OR Dog OR dogs OR canine OR canis OR felis)) AND ((TITLE-ABS-KEY(pyelonephrit* OR nephrit* OR pyelitis OR pyonephros*)) OR ((TITLE-ABS-KEY("Acute kidney injur*" OR "Acute kidney failure" OR "Acute Renal failure")) AND (TITLE-ABS-KEY(Infect* OR Bacteri*))))
