# Supplementary material for: Pyelonephritis in cats and dogs - a scoping review
Source: BMC Vet Res. 2026 Oct 26;22:635. doi: 10.1186/s12917-026-06042-1 (PMC13712058; doi:10.1186/s12917-026-06042-1)
Supplement: Supplementary file 3 [file 12917_2026_6042_MOESM3_ESM.pdf]

Britt Marie Bergquist and Isabelle Nesterud, SLU library

Second search on the 9th of January 2025.

Database: Biosis
Date: 2025-01-09

**Search**	**Terms**	**Result**
1	TS=(cat OR cats OR feline OR Dog OR dogs OR canine OR canis OR felis)	170,093
2	TS=((pyelonephrit* OR nephrit* OR pyelitis OR pyonephros*) OR ((“Acute kidney injur*” OR “Acute kidney failure” OR “Acute Renal failure”) AND (Infect* OR Bacteri*)))	23,184
3	1 AND 2	339
4	Year: 2020-	126

Database: CABI, Cab Abstracts
Date: 2025-02-04

**Search**	**Terms**	**Result**
1	TS=(cat OR cats OR feline OR Dog OR dogs OR canine OR canis OR felis)	405,775
2	TS=((pyelonephrit* OR nephrit* OR pyelitis OR pyonephros*) OR ((“Acute kidney injur*” OR “Acute kidney failure” OR “Acute Renal failure”) AND (Infect* OR Bacteri*)))	8,728
3	1 AND 2	1,448
4	Year: 2020-	216

Database: Medline via WoS
Date: 2025-02-04

**Search**	**Terms**	**Result**
1	TS=(cat OR cats OR feline OR Dog OR dogs OR canine OR canis OR felis)	608,510
2	TS=((pyelonephrit* OR nephrit* OR pyelitis OR pyonephros*) OR ((“Acute kidney injur*” OR “Acute kidney failure” OR “Acute Renal failure”) AND (Infect* OR Bacteri*)))	76,100
3	MHX=(Pyelonephritis OR Nephritis, Interstitial OR Pyelitis OR Pyonephrosis OR Acute Kidney Injury)	81,089

4	2 OR 3	128,091
5	1 AND 4	2,234
6	Year: 2020-	314

Database: Web of Science, Core collection (SCI-EXPANDED, SSCI, AHCI, ESCI)
Date: 2025-02-04

**Search**	**Terms**	**Result**
1	TS=(cat OR cats OR feline OR Dog OR dogs OR canine OR canis OR felis)	698,287
2	TS=((pyelonephrit* OR nephrit* OR pyelitis OR pyonephros*) OR ((“Acute kidney injur*” OR “Acute kidney failure” OR “Acute Renal failure”) AND (Infect* OR Bacteri*)))	68,770
3	1 AND 2	1,157
4	Year: 2020-	205

Database: Scopus
Date: 2025-02-04

**Search**	**Terms**	**Result**
1	TITLE-ABS-KEY(cat OR cats OR feline OR Dog OR dogs OR canine OR canis OR felis)	817,731
2	(TITLE-ABS-KEY(pyelonephrit* OR nephrit* OR pyelitis OR pyonephros*)) OR ((TITLE-ABS-KEY("Acute kidney injur*" OR "Acute kidney failure" OR "Acute Renal failure")) AND (TITLE-ABS-KEY(Infect* OR Bacteri*)))	130,456
3	1 AND 2	1,939
4	Year: 2020-	485
